# Dual-modal Fe-MOF-derived piezoelectric nanofibers enable combinatorial tumor suppression via tumor treating fields and magnetic hyperthermia

**DOI:** 10.1080/14686996.2026.2705817

**Published:** 2026-09-11

**Authors:** Shiqin Dai, Ahmed Nabil, Mitsuhiro Ebara

**Affiliations:** a Research Center for Macromolecules and Biomaterials, National Institute for Materials Science (NIMS), Tsukuba, Ibaraki, Japan; b Graduate School of Pure and Applied Sciences, University of Tsukuba, Tsukuba, Ibaraki, Japan; c Department of Materials Science and Technology, Tokyo University of Science, Tokyo, Japan; d Department of Biochemistry and Microbiology, Faculty of Pharmaceutical Sciences, Chulalongkorn University, Bangkok, Thailand

**Keywords:** Glioblastoma, tumor treating fields therapy, magnetic hyperthermia, MOF-derived porous carbon, nanofibers, combinatorial therapy

## Abstract

Glioblastoma (GBM) is among the most lethal primary brain tumors, owing to its highly invasive nature and pronounced resistance to conventional therapies. Emerging physical treatment modalities, such as tumor treating fields (TTFs), offer drug-free therapeutic alternatives. However, their clinical efficacy remains constrained by limited field strength and poor spatial control in deep brain regions. Moreover, a single physical stimulus is often inadequate to achieve sustained tumor suppression, highlighting the need for complementary therapeutic modalities. Herein, we report a multifunctional therapeutic platform in which iron-based metal-organic framework (Fe-MOF)-derived porous carbon is incorporated into piezoelectric polyacrylonitrile (PAN) nanofibers. This system enables ultrasound-triggered local electric field generation in combination with alternating magnetic field (AMF)-driven magnetic hyperthermia. The engineered nanofibers exhibited enhanced piezoelectric performance under ultrasound stimulation, alongside efficient heat generation under magnetic excitation. In vitro studies demonstrated pronounced suppression of GBM cell migration and induction of apoptosis, arising from the combinatorial effects of localized electric field stimulation and thermal therapy. Collectively, this work establishes a dual-modal therapeutic strategy that overcomes key limitations of standalone therapies and offers a promising platform for effective GBM treatment.

## IMPACT STATEMENT

Glioblastoma (GBM) remains one of the most aggressive and treatment-resistant primary brain tumors, with conventional therapies limited by high invasiveness and poor clinical outcomes. Herein, we present a multifunctional nanofibrous platform based on Fe-MOF-derived porous carbon embedded within piezoelectric polyacrylonitrile (PAN) fibers, enabling combinatorial physical therapy. The engineered composite fibers generate localized electric fields under ultrasound stimulation while simultaneously producing heat under alternating magnetic field (AMF) exposure, thereby overcoming the limited penetration depth and poor spatial controllability associated with single-modality treatments.

In vitro evaluations reveal significant tumor growth inhibition driven by the combined electro-thermal effects. This dual-functional platform integrates structural and functional design advantages, establishing a novel platform for coupling multiple physical stimuli. Collectively, it offers a promising and effective therapeutic approach for the treatment of deep-seated GBM.

## GRAPHICAL ABSTRACT

**Figure:**
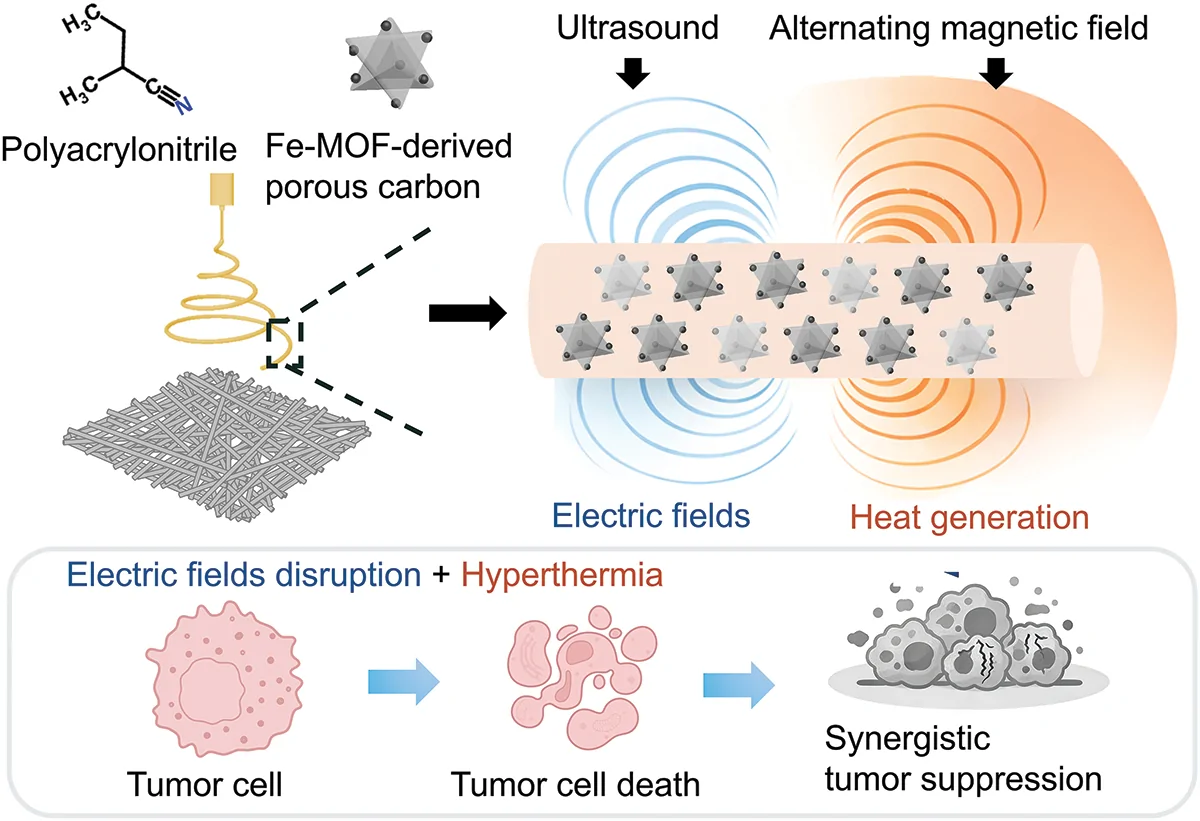

## 1. Introduction

Glioblastoma (GBM) is the most aggressive primary malignant tumor of the central nervous system, characterized by rapid proliferation, diffuse infiltration, and profound intratumoral heterogeneity [1–3]. The current standard of care consists of maximal safe surgical resection followed by temozolomide-based chemotherapy. However, the median survival of GBM patients remains approximately 12–18 months with a five-year survival rate below 10% [4,5]. Treatment failure is primarily attributed to intrinsic chemoradiotherapy resistance, the persistence of glioma stem cells, and the restrictive properties of the blood-brain barrier, which significantly impede effective drug delivery to deep-seated tumor regions [6–8]. These challenges underscore the urgent need for alternative therapeutic strategies capable of circumventing pharmacological limitations while maintaining anticancer effectiveness. Physical therapy has emerged as a viable non-pharmacological modality for GBM treatment. Among these modalities, tumor treating fields (TTFs) have been approved as an adjuvant therapy for newly diagnosed GBM and exert their therapeutic effects by applying low-intensity, intermediate-frequency alternating electric fields to disrupt mitotic spindle assembly [9–11]. However, conventional TTFs systems rely on externally affixed electrodes, resulting in substantial electric field attenuation with increasing tissue depth and limited precision in targeting intracranial tumors [12]. Recent studies have explored ultrasound-activated piezocatalytic therapy combined with TTFs-like electrical stimulation [13] and ferroelectric nanoparticle-mediated electric field amplification [14]. Nevertheless, both approaches depend on a single physical stimulus, which is frequently insufficient to achieve sustained tumor suppression in highly aggressive GBM [15]. Consequently, combinatorial strategies integrating multiple physical stimuli have gained considerable interest.

Ultrasound (US) has attracted increasing attention as a non-invasive energy source for biomedical applications owing to its deep tissue penetration, high spatiotemporal controllability, and favorable clinical safety profile [16]. Compared with optical or electromagnetic stimulation modalities, acoustic waves propagate efficiently through biological tissues with minimal attenuation, enabling precise energy delivery to deep-seated pathological sites [17]. Recent studies have demonstrated that US can be effectively coupled with piezoelectric nanomaterials to convert mechanical vibrations into localized electrical signals, thereby enabling wireless electrical stimulation without the need for implanted electrodes or external power sources. For instance, Wu and co-workers reported that ultrasound-activated piezoelectric SnS nanosheets generate localized electric fields capable of modulating cellular activity and inducing antitumor effects in deep tissues [18]. Similarly, Jin and co-workers demonstrated that an ultrasound piezoelectric energy harvester (U-PEH) can generate reactive oxygen species and bioelectrical stimulation, synergistically enhancing tumor treating fields and significantly improving antitumor efficacy [13]. Among various piezoelectric materials, polymeric systems have attracted particular interest for neural and intracranial applications because of their intrinsic flexibility and mechanical compatibility with soft tissues [19]. In this context, polyacrylonitrile (PAN) has emerged as a promising candidate owing to its favorable biocompatibility, chemical stability, and capacity to form nanofibers structures via electrospinning, which significantly increases the effective surface area and enhance its piezoelectric property [20,21]. It has been reported that electrospinning induces molecular chain alignment and dipole orientation in PAN nanofibers, resulting in unexpectedly high piezoelectric coefficients [22]. Furthermore, PAN-based piezoelectric nanofibers can efficiently convert sound and mechanical noise into usable electrical energy through piezoelectric effects, revealing their potential for self‑powered acoustic energy harvesting applications [23]. However, despite these advantages, the inherently modest piezoelectric coefficients of polymeric materials result in comparatively weak electrical outputs under US stimulation. The induced electric fields are insufficient to achieve therapeutic intensities comparable to those employed in tumor treating fields bioelectric stimulation. This limitation consequently constrains their therapeutic effectiveness against highly aggressive malignancies like GBM [24].

Therefore, strategies that simultaneously enhance the piezoelectric performance of polymeric materials and incorporate multiple physical modalities are essential to realizing combinatorial therapy and strengthening antitumor efficacy. Hyperthermia induces a localized temperature elevation, thereby sensitizing tumor cells to treatment. This thermal effect disrupts protein homeostasis, impairs DNA repair mechanisms, and remodels the tumor microenvironment [25]. Magnetic hyperthermia driven by alternating magnetic fields (AMF) offers deep, contactless, and spatially controllable heat generation, and has shown encouraging outcomes in recent preclinical research on gliomas [26]. In our previous work, we established chemo-thermal synergy by integrating magnetic nanoparticles with thermally responsive drug delivery systems. Specifically, we developed a Diels-Alder-based platform using magnetic nanoparticles that enabled AMF-triggered drug release, demonstrating the feasibility of thermo-responsive chemotherapy [27,28]. Building upon this strategy, we further designed nanofiber-based systems capable of sustained and combinatorial drug delivery, yielding enhanced synergistic therapeutic efficacy [29]. In addition, we developed locally implantable nanofiber meshes for the sustained release of temozolomide, enabling effective thermo-chemotherapy for glioblastoma treatment [30]. Despite these advances, overall therapeutic efficacy remains constrained by insufficient drug penetration within heterogeneous tumor regions and by drug-associated adverse effects [31]. Accordingly, there remains a need to develop more integrative therapeutic platforms that can further augment the combinatorial efficacy of a coordinated multi-modal systems while avoiding the use of pharmacological agents. Iron-based metal-organic framework (Fe-MOF)-derived porous carbon provides a versatile platform for constructing multimodal nanocomposites owing to its compositional tunability and structural diversity [32,33]. Upon high-temperature carbonization, Fe-MOF can be converted into Fe-MOF-derived porous carbon (MPCs) matrices embedded with Fe_3_O_4_ nanoparticles [34], endowing the resulting materials with both magnetic loss capability for efficient hyperthermia and enhanced piezoelectric response for electric field generation. When MPCs are integrated into piezoelectric nanofibers, the strong interfacial coupling between the MOF-derived porous carbon and polymer chains promotes dipole alignment and stress-induced charge separation, resulting in an enhanced piezoelectric response under ultrasound (US) excitation [35].

Herein, we report an MPCs/PAN nanofibers platform that integrates ultrasound-activated piezoelectric therapy with magnetic hyperthermia within a single material system (Scheme 1). This dual-functional platform enables combinatorial in situ electric field generation and localized heat production, effectively overcoming the intrinsic limitations of standalone physical therapies. The strong interfacial coupling between MPCs and PAN nanofibers not only enhances dipole alignment and stress-induced charge separation under US stimulation but also provides efficient magnetic loss for controllable hyperthermia, enabling precise energy delivery to deep tissue targets. In vitro studies demonstrate that this integrated platform effectively inhibits GBM cell migration, induces apoptosis, and achieves superior antitumor efficacy compared with monotherapy approaches.

**Scheme 1.**
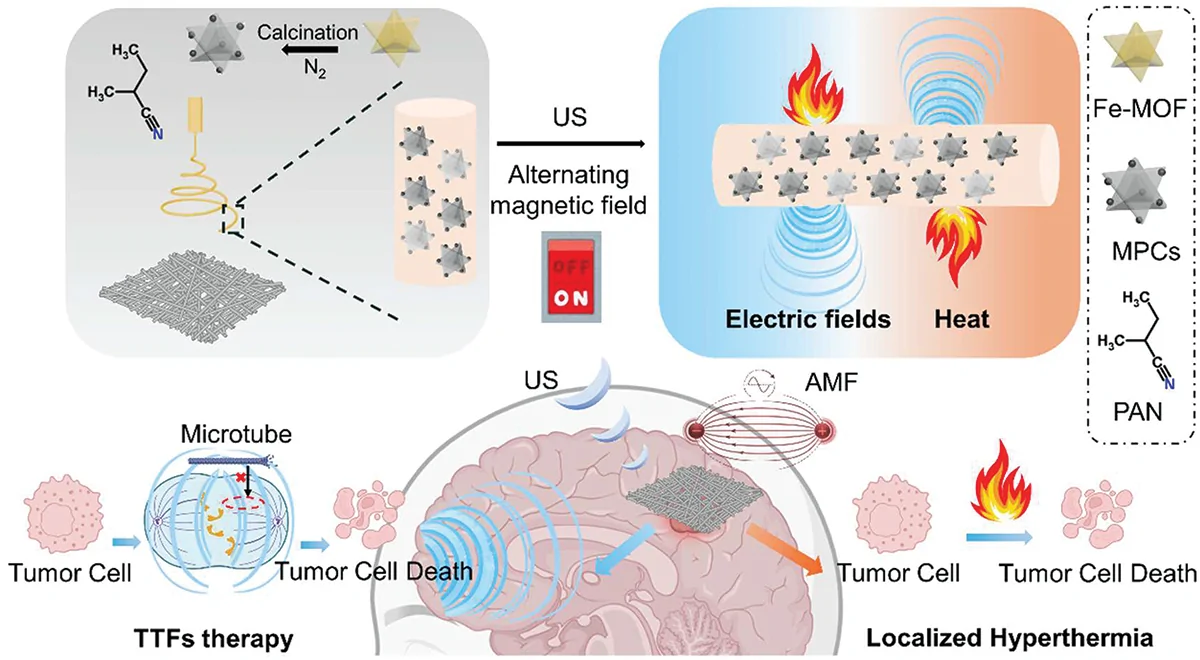
Schematic illustration of the design and therapeutic mechanism of MPCs/PAN nanofibrous platform enabling combinatorial tumor suppression via tumor treating fields and magnetic hyperthermia.

## 2. Materials and methods

### 2.1. Materials and reagents

Polyacrylonitrile (PAN, Mw = 1,500,000), Pluronic F-127 (F-127), and iron(III) chloride hexahydrate (FeCl_3_·6H_2_O) were obtained from Sigma-Aldrich, CO. (St. Louis, MO, USA), while the 2‑Amino‑1,4‑benzenedicarboxylic acid (NH_2_-BDC) was supplied by Tokyo Chemical Industry CO., Ltd. (Tokyo, Japan). N,N-Dimethylformamide (DMF) was purchased from Fujifilm Wako Pure Chemical CO. (Osaka, Japan). Fetal bovine serum (FBS) was provided by Tocris Bioscience Inc. (Minneapolis, MN, USA). Trypsin, penicillin, and streptomycin, acetic acid, and 3-(4,5-dimethylthiazol-2-yl)-2,5-diphenyl tetrazolium bromide (MTT) assay were obtained from Nacalai Tesque, Inc. (Kyoto, Japan). T98G human glioblastoma cell line was obtained from the RIKEN BioResource Research Center Cell Bank (RIKEN BRC, Ibaraki, Japan).

### 2.2. Synthesis of MPCs and MPCs/PAN composite fibers

Fe-MOF was synthesized via a modified hydrothermal method. Briefly, 1.6 g of F-127 was dissolved in 66.7 mL of deionized water, followed by addition of 8.3 mL of FeCl_3_·6H_2_O solution (0.38 M). The mixture was stirred for 1 h, after which 1.1 mL of acetic acid was introduced. Following an additional 1 h of stirring, 0.3 g of NH_2_-BDC was added to the solution. The resulting mixture was stirred for a further 2 h and subsequently transferred into a Teflon-lined autoclave for hydrothermal crystallization. The dark-brown Fe-MOF solid was collected, washed repeatedly with DMF and ethanol, and centrifuged to remove residual reactants and surfactants. The collected solid was dried in vacuum oven at 60°C overnight. Carbonization was performed at 400, 500, and 600°C with a heating rate of 2°C min^−1^ for 40 min under a N_2_ atmosphere to yield MPCs. The final products were cooled to room temperature and stored for further use. For nanofibers fabrication, MPCs were dispersed in 3 mL of DMF at concentrations corresponding to 5, 10 and 15 wt.% relative to PAN. The dispersions were sonicated for 20 min to ensure homogeneous distribution of the particles. Subsequently, 10 wt.% amounts of PAN powder was added and stirred at 45°C for 2 h to form uniform electrospinning solutions. Electrospinning was performed by using an electrospinning machine Nanon-01A was used at a voltage of 25 kV (MECC Co., Ltd., Fukuoka, Japan). The spinning solution was delivered through a 27-gauge needle positioned 15 cm from an aluminum foil collector. The flow rate was maintained at 0.3 mL h^−1^ to produce uniform MPCs/PAN nanofibers.

### 2.3. Characterizations of MPCs and MPCs/PAN composite fibers

The morphology and elemental distribution of MPCs were characterized by transmission electron microscopy (TEM, JEM-2100F2, JEOL Ltd., Japan). The surface morphology of MPCs/PAN composite nanofibers was examined by scanning electron microscopy (SEM, SU8230, Hitachi, Japan). Elemental composition and spatial distribution were further analyzed by energy-dispersive X-ray spectroscopy (EDS). SEM observations were conducted at an accelerating voltage of 10 kV with a working distance of approximately 3 mm. Prior to imaging, all samples were coated with a 3 nm platinum layer to enhance conductivity. The hydrodynamic diameter of MPCs was determined by dynamic light scattering (DLS, ELSZ-2000ZS, Otsuka Electronics, Japan). Fourier transform infrared (FT-IR) spectra were acquired using an FT-IR spectrophotometer (IRAffinity-1S, Shimadzu, Japan). Crystallographic structure and phase composition were analyzed by X-ray diffraction (XRD, MiniFlex, Rigaku Corporation, Tokyo, Japan) over a 2θ range of 3–80° at a scanning rate of 10° min^−1^ under operating conditions of 40 kV and 15 mA. The elemental composition and chemical states of the Fe-MOF were further investigated using hard X-ray photoelectron spectroscopy (HAXPES/XPS, ULVAC-PHI, Inc., Chigasaki, Japan). Image analysis was performed using ImageJ software (version 1.54p, National Institutes of Health, USA) to quantify nanofiber diameters.

### 2.4. Piezoelectric profiles for nanofibers

Ultrasound stimulation was applied using an ultrasonic generator (KM-1200, Nanjing Guogong Ultrasonic Technology Co., Ltd., Nanjing, China) equipped with a 40 kHz ultrasound probe. To measure the output voltage of nanofibers, the nanofibers were sandwiched between two copper (Cu) electrodes (15 mm × 15 mm × 1 mm each) and encapsulated between top and bottom polyurethane (PU) insulating layers to minimize environmental interference. The copper electrodes were connected to an oscilloscope (DS1102 Z-E, Rigol Technologies, Inc., Beijing, China) through a voltage probe (PVP3150, Rigol Technologies, Inc., Beijing, China) with a 10-megohm input impedance to record the output voltage. The electric field strength (E) was calculated by dividing the potential difference under working load (U) by the separating distance (d) between plates.

### 2.5. Heating profiles for nanofibers

The heat-generating performance of nanofiber samples (1 × 1 cm^2^) were evaluated under alternating magnetic field (AMF) irradiation. A custom-designed copper coil was used to accommodate the nanofibers, which were then exposed to an AMF generated by an EASYHEAT 8310 system (Ambrell Co., Ltd., Rochester, NY, USA) operating at a frequency of 274 kHz and current amplitudes of 500, 600, and 700 A. The AMF was generated using a custom-built induction coil with a geometry of *n* = 3 turns and a coil length of l = 0.5 cm. The magnetic field strength (H) was calculated according to H = nI/l (I = current), the field strengths corresponding to 500, 600, and 700 A coil currents are 300, 360, and 420 kA m^−1^, respectively. Temperature evolution during AMF exposure was monitored at predefined time intervals using an infrared thermal imaging camera (FLIR CPA-E6, FLIR Systems Japan K.K., Tokyo, Japan). The specific absorption rate (SAR) was calculated from the initial linear slope of the temperature-time heating curve according to the equation: SAR = (C × m_sample_ / m_MPC_) × (dT/dt)|_*t* = 0_, where C is the specific heat capacity of the MPCs/PAN nanofibers, m_sample_ is the total weight of the nanofibers, *m*_MPC_ is the weight of MPCs within the nanofibers, and dT/dt|_*t* = 0_ is the initial rate of temperature rise (°C s^−1^) determined from the linear region of the temperature-time curve (0–1 min) under AMF stimulation at 360 kA m^−1^ and 274 kHz.

### 2.6. Magnetization curve

The magnetization-magnetic field (M-H) curve of the MPCs was measured under a DC magnetic field using a physical property measurement system (PPMS, Quantum Design Inc., CA, US) at room temperature. MPCs nanoparticles were loaded into a plastic sample tube and placed in the machine.

### 2.7. In vitro experiments

T98G human glioblastoma cells were used throughout this study. Cells were cultured in RPMI 1640 medium supplemented with 10% fetal bovine serum (FBS) and 1% penicillin-streptomycin under standard incubation conditions (37°C, 5% CO_2_, MCO-170AICUVH, PHC Holdings Corporation, Tokyo, Japan). Cells were maintained in 100 mm culture dishes for 3 days until reaching appropriate confluence. For experimental assays, cells were detached using 0.25% (w/v) trypsin and seeded into 35 mm culture dishes at a density of 1.0 × 10^6^ cells per dish in 2 mL of growth medium, followed by 24 h incubation to allow cell attachment. Subsequently, PAN fibers and MPCs/PAN composite fibers were then dispersed in the culture medium, and the cells were exposed to US stimulation for 8 h. Cell viability was assessed 24 h later using the MTT assay. For cell migration analysis, T98G cells were seeded in six-well plates at a density of 1 × 10^6^ cells per well and cultured until approximately 90% confluence. A linear scratch was introduced using a 200 μL pipette tip, and detached cells were removed by washing with phosphate-buffered saline (PBS). Cells were subsequently incubated in fresh medium, and wound closure was monitored at 0 and 24 h by optical microscopy. The cell migration rate was calculated as follows: Cell migration rate = (scratch distance at 0 h – scratch distance at 24 h) / scratch distance at 0 h. Intracellular reactive oxygen species (ROS) levels were measured using a ROS assay kit (Cat. No. R253, Dojindo Laboratories, Kumamoto, Japan) according to the manufacturer’s instructions and analyzed using a fluorescence microscope (Eclipse Ti2-E microscope, Nikon Corporation, Tokyo, Japan). Apoptosis was evaluated using an Annexin V-DY-634/PI Apoptosis Staining Kit (Cat. No. ab214484, Abcam, Cambridge, UK) according to the manufacturer’s protocol. Quantitative analysis was performed using a BD Accuri C6 Plus Flow Cytometer (BD Biosciences, Franklin Lakes, NJ, USA).

### 2.8. Statistical analysis

All experiments were performed in triplicate. Data are presented as mean ± standard deviation (SD). Statistical comparisons were conducted using Student’s t-test and one-way analysis of variance (ANOVA) followed by Tukey’s post hoc test, performed in Origin version 9.0 software (OriginLab Corporation, Northampton, MA, USA). Differences were considered statistically significant at *p* < 0.001 (***), *p* < 0.01 (**), and *p* < 0.05 (*).

## 3. Results and discussion

### 3.1. Characterization of Fe-MOF and MPCs

To characterize the chemical structure, morphology, and elemental composition of the Fe-MOF, FT-IR, XRD, and XPS analyses were performed. As shown in Figure 1(A), FT-IR spectrum revealed absorption peaks of Fe-MOF at 3560 cm^−1^ and 3033–2966 cm^−1^ were attributed to O-H stretching vibrations and aromatic C-H (sp^2^) stretching, respectively. Absorption peaks at 1676 and 1355–1436 cm^−1^ were assigned to C-H bending vibrations as well as the asymmetric and symmetric stretching modes of carboxylate (COO^−^) groups, respectively. Peaks in the region of 671–549 cm^−1^ were associated with Fe-O vibrations, indicating coordination between the metal ions and the ligand. Notably, the C=O-related vibrational band of the linker from 1683 cm^−1^ toward lower wavenumbers at 1676 cm^−1^ suggests the successful coordination between the carboxylate groups and Fe^3+^ ions, confirming the formation of the Fe-MOF framework [36]. The XRD pattern of Fe-MOF exhibited diffraction peaks at approximately 10.14°, 12.42°, 16.06°, 17.64°, 19.82°, 24.48°, 26.56°, and 29.4°, consistent with the reported pattern for Fe-MOF, confirming its successful synthesis (Figure 1(B)) [37]. The XPS spectrum confirmed the presence of Fe, O, C, and N in agreement with the expected composition of the Fe-MOF constructed from Fe and NH_2_-BDC linkers (Figure 1(C)). The N signal is attributed to the amino functionality of the ligand, supporting its incorporation into the framework. High-resolution XPS analysis of the Fe 2p region provided further insight into the chemical state of Fe species (Figure 1(D)) [38]. The two main peaks centred at 710.3 eV and 723.5 eV corresponded to Fe 2p_3/2_ and Fe 2p_1/2_, respectively. These binding energies are characteristic of Fe species in a coordination environment. The absence of additional peaks associated with impurity phases suggested that Fe was predominantly present in a coordinated state within the framework [39]. Collectively, the FT-IR, XRD, and XPS data corroborated the successful coordination between Fe^3+^ ions and NH_2_-BDC ligands, confirming the formation of the Fe-MOF structure via hydrothermal synthesis.

**Figure 1.**
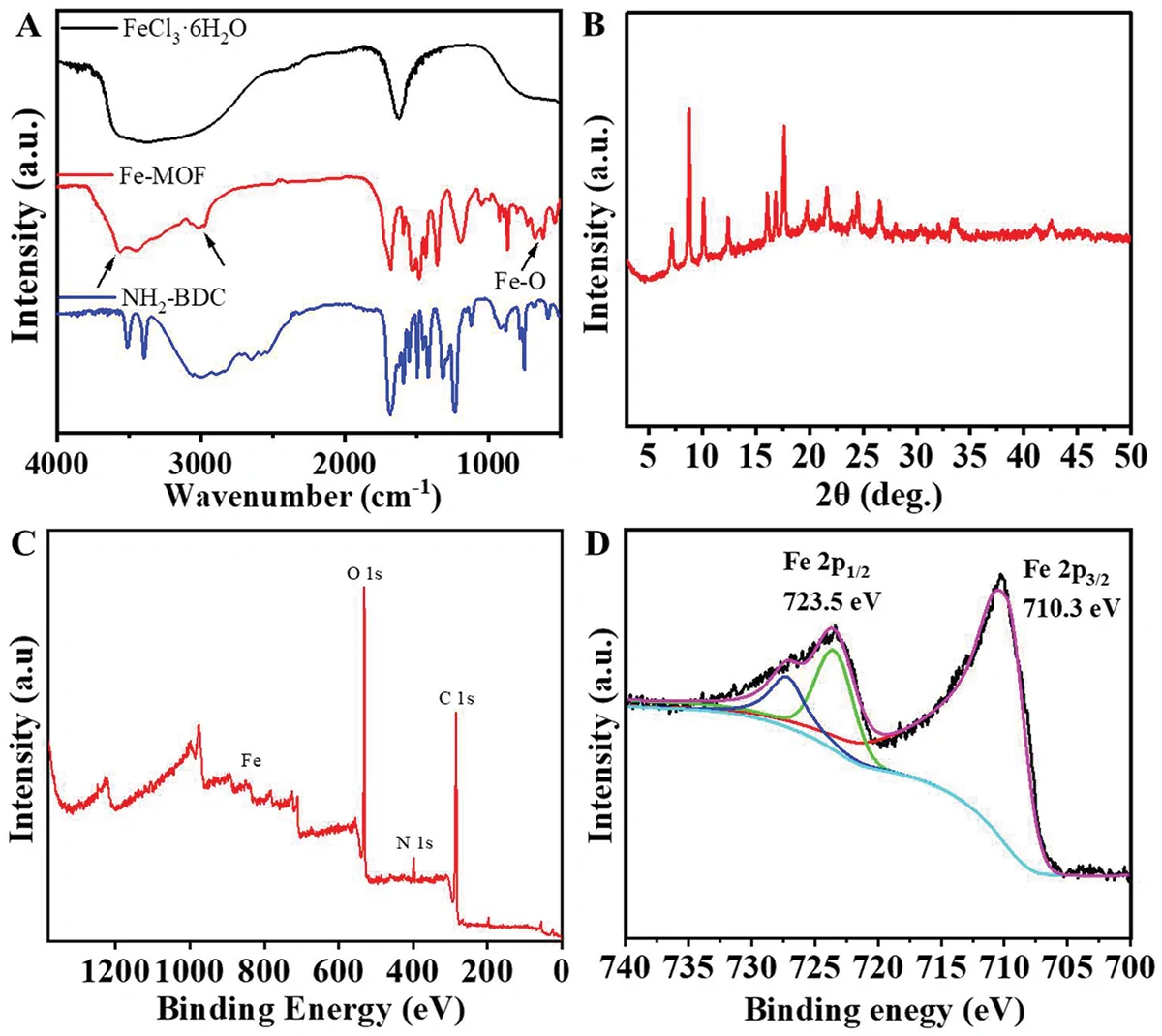
(A) FT-IR spectra, (B) XRD patterns, (C) XPS spectroscopy, (D) High-resolution XPS spectroscopy of Fe-MOF.

DLS, TEM and EDS were employed to characterize the construction of MPCs with Fe_3_O_4_ nanoparticles embedded within a carbon matrix. DLS analysis showed that the Fe-MOFs had a particle size of 123 nm before calcination at 500°C (Figure S1A), which slightly decreased to 100 nm for the resulting MPCs, indicating that the framework largely retained its structural integrity after calcination (Figure S1B). TEM imaging revealed that the MPCs consisted of mesoporous carbon nanoparticles with an average diameter of 105 nm. Fe_3_O_4_ nanocrystals were uniformly distributed across their surfaces (Figure 2(A)). HRTEM image confirmed the lattice fringes of Fe_3_O_4_ with a spacing of 0.24 nm, consistent with the (311) crystal plane of cubic Fe_3_O_4_ (Figure 2(B,C)). This result attested to well-defined crystallinity of the Fe_3_O_4_ phase [40]. STEM images showed the distribution of Fe_3_O_4_ within carbon matrix (Figure 2(D)). Furthermore, EDS mapping demonstrated that Fe and O elements were predominantly localized within the nanoparticle domains, consistent with the spatial distribution of Fe_3_O_4_, while C and N were homogeneously distributed throughout the structure, consistent with the carbon matrix and nitrogen-containing functionalities. Taken together, these results showed the successful synthesis of MPCs with Fe_3_O_4_ nanoparticles effectively incorporated into the mesoporous carbon framework.

**Figure 2.**
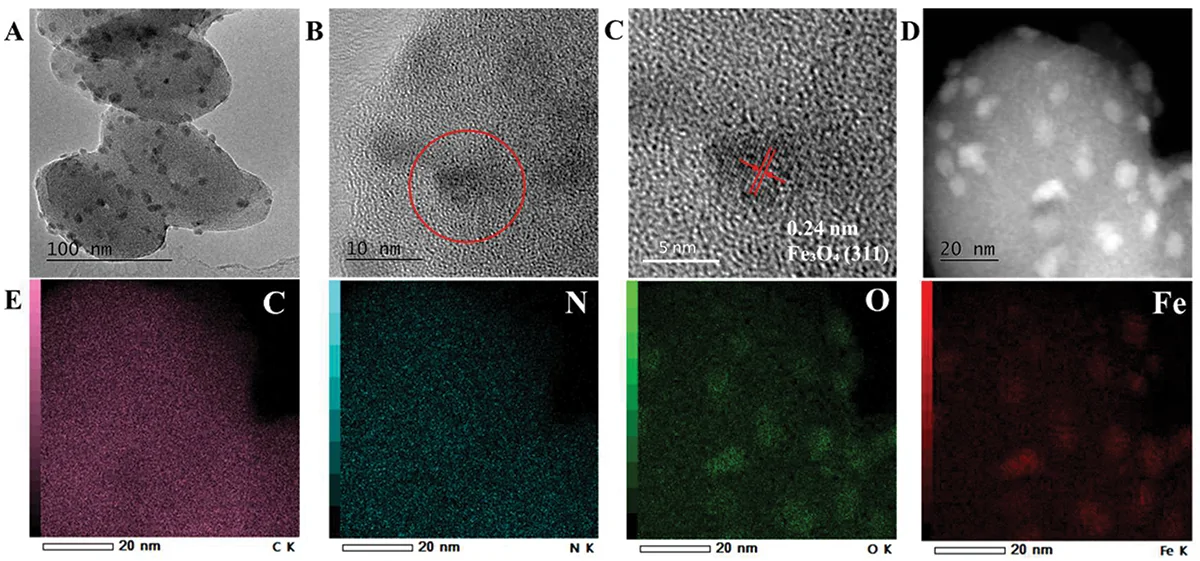
(A) TEM, (B-C) HRTEM, (D) STEM, (E) EDS of MPCs.

### 3.2. Characterization of MPCs/PAN composite nanofibers

To evaluate the effect of calcination temperature on Fe-MOF, XRD analysis was performed. During N_2_-atmosphere calcination of the Fe-MOF, the carbonaceous matrix derived from the organic linker generates a local reducing environment via the boudouard equilibrium. This reducing environment drives reduction of the initial Fe^3+^ precursor to phase-pure Fe_3_O_4_ [41]. As shown in Figure 3(A), the diffraction patterns exhibited structural evolution with increasing calcination temperature. For the Fe-MOF calcined at 500°C, several distinct diffraction peaks at approximately 30.2°, 33.1°, 35.6°, 43.7°, and 57.0° were indexed to the (220), (311), (400), (511), and (440) planes of Fe_3_O_4_ (Fe_3_O_4_, JCPDS Card No. 019–0629), respectively. These results confirmed the formation of Fe_3_O_4_ nanoparticles derived from the Fe centres of the MOF precursor. In addition, a broad diffraction peak centred around 24° was also observed, corresponding to the (002) plane of amorphous or turbostratic carbon, originating from the carbonization of the organic ligands [42]. By contrast, the sample calcined at 400°C exhibited no distinct carbon (002) peak and only weak reflections assignable to the (220), (511), and (440) planes of Fe_3_O_4_, indicative of insufficient carbonization and incomplete Fe_3_O_4_ formation. In contrast, a new diffraction peak at 36° in the MPCs-600°C sample was consistently assigned to the (111) reflection of Iron(II) oxide (FeO, JCPDS No. 89–0687), with a companion (200) reflection observed at 42°. Considering the balance between Fe_3_O_4_ formation and carbon framework integrity, MPCs calcined at 500°C were selected for subsequent experiments. FT-IR analysis was performed to confirm the formation of the composite nanofibers. As shown in Figure 3(B), FT-IR spectra of the PAN nanofibers exhibited characteristic absorption bands at 2243 cm^−1^ corresponding to the C≡N stretching vibration, and bands near 2939, 1456 and 1350 cm^−1^ attributed to C-H stretching in the backbone of the PAN nanofibers. The Fe-O band at 769 cm^−1^ was from MPCs. The MPCs/PAN nanofibers exhibited characteristic peaks from both PAN and MPCs, indicating the coexistence of the two components. The morphology of the MPCs/PAN composite nanofibers was further investigated by SEM. As shown in Figure 3(C), the electrospun PAN fibers maintained uniform fibrous morphology after the incorporation of MPCs, exhibiting a smooth and interconnected network structure. Furthermore, SEM images of 5 wt.% MPCs/PAN (Figure S2A), 10 wt.% MPCs/PAN (Figure S2B), and 15 wt.% MPCs/PAN nanofibers (Figure S2C) at both 2k and 5k magnifications confirmed that all samples exhibited uniform, bead-free fibrous morphologies, with quantified average fiber diameter of 400–500 nm. At higher magnification, MPCs were observed partially embedded within the nanofibers, indicating their successful incorporation into the electrospun matrix (Figure 3(D)). This structure provides interfacial contact between the MPCs and the polymer chains, which is expected to facilitate efficient interfacial interactions within the composite system. EDS elemental mapping was performed to verify the elemental distribution within the composite nanofibers (Figure 3(E)). The Fe and O signals originate from the Fe_3_O_4_ nanoparticles, while C and N correspond to the PAN matrix. The homogeneous distribution of these elements further confirmed the effective integration of MPCs into the PAN nanofibers, forming a well-integrated composite architecture.

**Figure 3.**
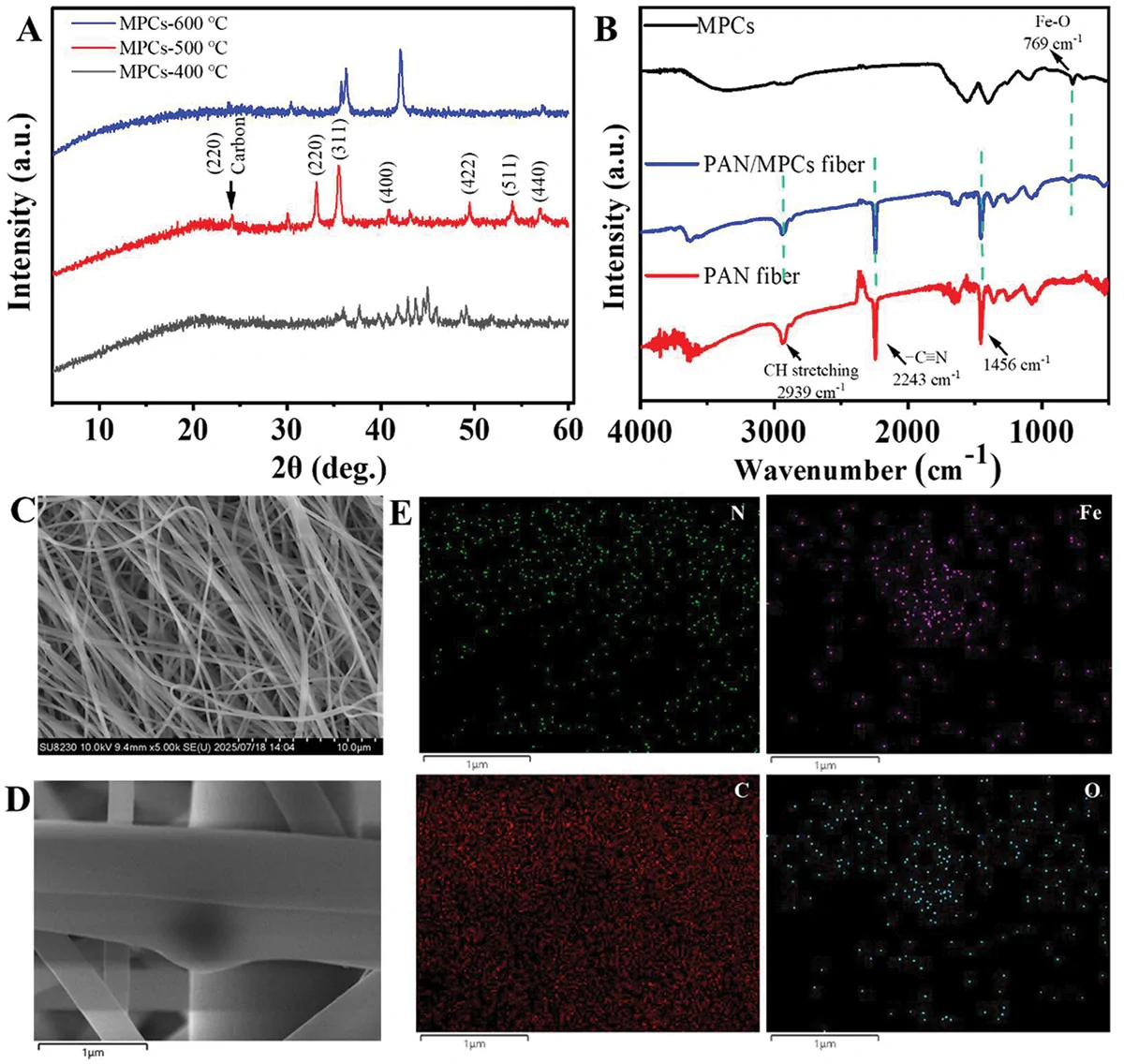
(A) XRD of MPCs prepared under different calcination temperatures. (B) FT-IR of MPCs, PAN nanofibers, MPCs/PAN composite nanofibers. (C-D) SEM, (E) EDS mapping of MPCs/PAN composite nanofibers.

### 3.3. Characterizations of hyperthermia property

The magnetic hyperthermia performance of the MPCs/PAN composite nanofibers was evaluated upon AMF exposure. As shown in Figure 4(A), the 15 wt.% MPCs/PAN nanofibers exhibited a temperature increase upon prolonged AMF irradiation, demonstrating efficient heat generation of the MPCs. To further investigate the effects of MPCs loading fraction and field intensity on hyperthermia performance, composite nanofibers containing 5 wt.%, 10 wt.%, and 15 wt.% MPCs/PAN fibers were exposed to AMF at current amplitudes of 500, 600, and 700 A (274 kHz), and temperature profiles were recorded over 10 min (Figure 4(B-D)). Under 500 A, all fibers remained below the mild hyperthermia range (42–45 °C), indicating insufficient heating for therapeutic application (Figure 4(B)). Upon increasing the field strength to 600 A, a substantial improvement in heating performance was observed (Figure 4(C)). In particular, the 15 wt.% MPCs/PAN nanofibers reached temperatures exceeding 45°C, potentially posing a risk of damage to healthy tissue. In contrast, the 10 wt.% MPCs/PAN nanofibers reached 42°C, remaining within the desired therapeutic window for magnetic hyperthermia. Further increasing the magnetic field strength to 700 A produced only marginal temperature elevation (Figure 4(D)). Accordingly, 10 wt.% MPCs/PAN composite nanofibers actuated under an AMF of 600 A and 274 kHz were selected as the optimal parameters for subsequent investigations, as this condition afforded an optimal balance between hyperthermic efficacy and biosafety. It should be noted that IR thermography was performed on dry nanofiber membranes, which likely overestimates the actual thermal exposure of cells in aqueous medium due to the higher heat capacity and convective dissipation of the liquid environment. Therefore, these measurements should be regarded as upper-bound estimates. Nevertheless, the significantly elevated apoptosis rate of 85.5% under combined US and AMF stimulation confirms effective tumor cell death, demonstrating that the combinatorial therapeutic effects remain valid under physiological conditions. To further elucidate the origin of the observed heating behaviour, the magnetic properties of MPCs calcined at 500°C were characterised at room temperature using a physical property measurement system. The nanoparticles exhibited a saturation magnetization (Ms) of 21.97 emu g^−1^ at an applied field of 1 T with negligible hysteresis, confirming superparamagnetic behaviour (Figure S3) [43]. The SAR values of MPCs/PAN nanofibers (2.15–3.19 W g^−1^) were lower than those of Fe_3_O_4_ nanoparticles (33–130 W g^−1^) [44], which is primarily attributed to two factors. First, the SAR was normalized to the total composite mass rather than the magnetic component alone, and the non-magnetic PAN matrix substantially diluted the mass-normalized heating efficiency. Second, the physical immobilization of MPCs within the solid polymer matrix likely suppressed the Brownian relaxation pathway. The magnetothermal heating is likely dominated by Néel relaxation based on the superparamagnetic characteristics and nanoscale size of the MPCs, though further alternating current susceptibility measurements are needed to confirm this. This superparamagnetic character enables efficient heat generation under AMF, supporting the application of MPCs in magnetic hyperthermia. Importantly, this heating efficiency is intrinsic to a multifunctional nanofibers that concurrently enables piezoelectric activity under ultrasound, a dual modality unattainable with bare magnetite.

**Figure 4.**
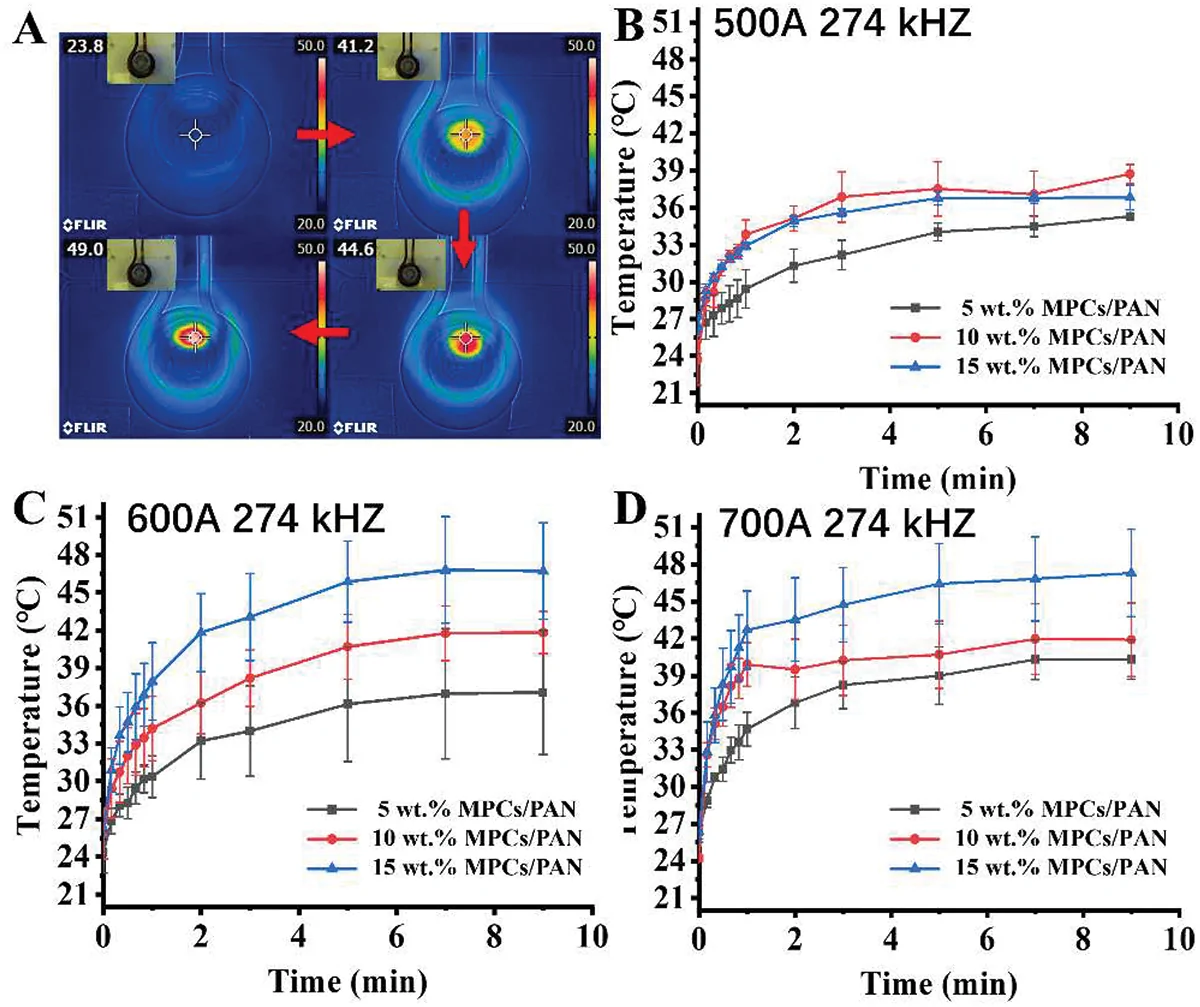
(A) Representative infrared thermal images of MPCs-loaded PAN fibers with AMF irradiation. (B-D) Heating profiles of MPCs/PAN fibers with relative 5 wt.%, 10 wt.%, and 15 wt.% MPCs contents during AMF application (500A, 600A, 700A). The data were obtained from three independent replicates (*n* = 3).

### 3.4. Characterizations of piezoelectric property

When MPCs are integrated into piezoelectric nanofibers, interfacial coupling between MPCs and polymer chains promotes dipole alignment and stress-induced charge separation. This effect results in an enhanced piezoelectric response under US excitation [35]. The piezoelectric performance of the MPCs/PAN composite nanofibers under US stimulation was evaluated using a sandwich-structured device configured for output voltage measurement (Figure 5(A)). The nanofibers were electrically connected via copper electrodes on both sides, while the outer PU layers provided insulation to minimize external interference. Mechanical oscillations induced by the US stimulation generate periodic stress on the piezoelectric nanofibers, the resulting deformation-induced dipole reorientation within the fiber matrix produces an alternating voltage output [45]. The output voltages of PAN, 10 wt.% MPCs/PAN, and 15 wt.% MPCs/PAN nanofibers were recorded under US stimulation (Figure 5(B)). PAN nanofibers exhibited a relatively low piezoelectric output, generating a voltage of 0.12 V. The 10 wt.% MPCs/PAN and 15 wt.% MPCs/PAN nanofibers achieved peak voltages of 0.20 V and 0.24 V, respectively. Furthermore, the thickness of MPCs/PAN fibers is 0.0878 cm, representing the electrode separation distance in the sandwich measurement configuration (Figure S4). This corresponded to a local electric field strength of 2.3 V cm^−1^ and 2.7 V cm^−1^. This estimated field strength of 2.3 V cm^−1^ and 2.7 V cm^−1^ aligned with the established therapeutic window for TTFs therapy (1–3 V cm^−1^), consistent with previously reported values for effective tumor growth inhibition. These results demonstrated the effective role of MPCs incorporation in augmenting the piezoelectric response of the composite system. In addition, the local electric field undergoes attenuation as it propagates through the biological medium, decreasing from 2.7 V cm^−1^ to approximately 1.4 V cm^−1^ at a medium thickness of 4 mm (Figure S5). The residual field strength remains within the established therapeutic window for TTFs therapy. These results suggested that the achieved electric field strength was sufficient to reach therapeutic intensities, supporting the potential of this platform for drug-free electrical anti-tumour therapy.

**Figure 5.**
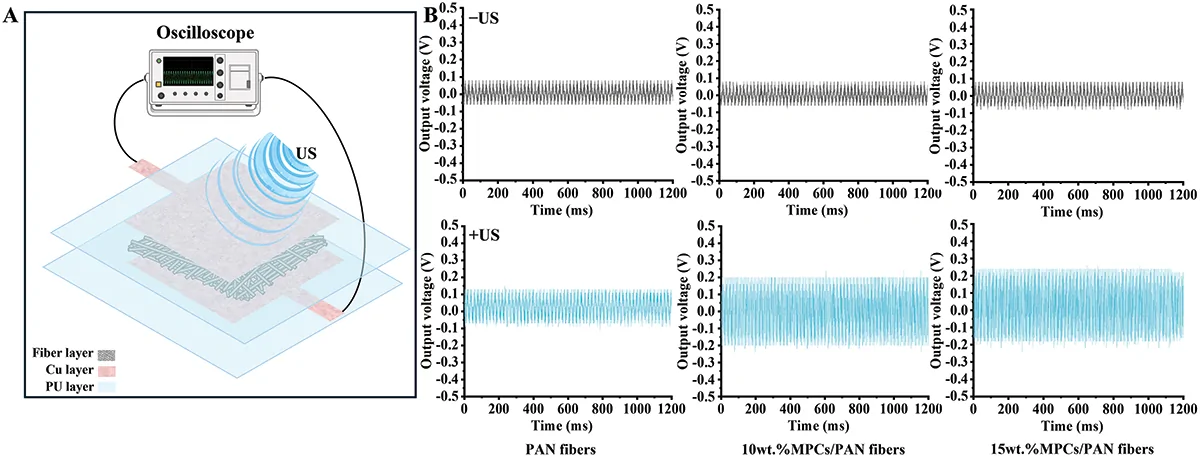
(A) Schematic diagram of the sandwich structure for measuring output voltage, (B) output voltage of PAN nanofibers, 10 wt.% MPCs/PAN nanofibers, 15 wt.% MPCs/PAN nanofibers under the stimulation of US.

### 3.5. In vitro experiments

To assess the effect of the treatment on cellular motility, an in vitro scratch assay was performed (Figure 6(A)). The untreated control group exhibited a gap closure rate of 0.42, indicating active cell migration. Also, after treating cells with US or AMF, there was no significant difference from the control group, indicating the effects of AMF and US were negligible. Notably, the 10 wt.% MPCs/PAN nanofibers under the stimulation of US and AMF significantly restricted cell migration relative to US stimulation alone. The cell migration rate decreased from 0.19 to 0.13, confirming the enhanced inhibitory effect of the dual-modal treatment over either US or AMF stimulation alone (Figure 6(B)). By applying US and AMF sequentially rather than simultaneously, the two modalities do not co-exist in time, and therefore no synergistic heating effect is generated during combined treatment. To elucidate the mechanism underlying AMF-mediated and US-mediated cytotoxicity, intracellular ROS levels were assessed using a fluorometric ROS assay kit. As shown in Figure S6, ROS levels remained at baseline in the US-only and US+MPCs/PAN groups, confirming that ultrasound stimulation alone does not induce oxidative stress under these conditions. ROS elevation was detected exclusively in the AMF+MPCs/PAN group and US+AMF+MPCs/PAN group, attributable to AMF-driven hyperthermia. This thermal effect perturbed the mitochondrial electron transport chain, promoting superoxide anion generation [46]. Thermally induced oxidative stress thus constituted a key cytotoxic mechanism of the combined treatment. Consistent with these ROS findings, cell viability assays confirmed the therapeutic efficacy of the MPCs/PAN composite nanofibers (Figure 6(C)). Cells exposed to US alone, US+AMF, and nanofibers alone maintained a relative viability of above 0.92, demonstrating negligible cytotoxicity under these conditions. However, when external stimuli were introduced, a stepwise reduction in cell viability was observed with increasing therapeutic complexity. The US +10 wt.% MPCs/PAN nanofibers group reduced cell viability to 0.80, indicating therapeutic contribution of the TTFs therapy. Upon integration of AMF stimulation, a pronounced enhancement in therapeutic efficacy was observed. In contrast, the US + AMF +10 wt.% MPCs/PAN fibers group reduced relative cell viability to 0.31. Notably, no significant difference in cell viability was observed between the AMF + PAN and PAN alone groups, indicating that AMF exposure in the absence of magnetic components produces no appreciable thermal effect. This confirms that magnetothermal hyperthermia is strictly dependent on the presence of MPCs, and that the cytotoxic response observed in the AMF +10 wt.% MPCs/PAN group is exclusively attributable to MPC-mediated heating rather than any non-specific effect of the applied field. In addition, flow cytometry analysis using Annexin V-FITC/PI double staining was performed to quantitatively evaluate the apoptotic response of tumor cells under different treatment conditions on tumor cells (Figure S7). In the untreated control groups (−US −AMF), both the control and 10 wt.% MPCs/PAN nanofiber groups exhibited high cell viability, with total apoptosis rates of only 0.84% and 0.11%, respectively, confirming negligible cytotoxicity of the nanofibers alone. Upon AMF stimulation alone (−US +AMF), the control group showed negligible apoptosis, whereas the 10 wt.% MPCs/PAN nanofiber group demonstrated a significantly elevated apoptotic rate of 27.7%. Strikingly, under combined US and AMF treatment (+US +AMF), the control group exhibited a total apoptosis rate of 7.60%, while the 10 wt.% MPCs/PAN nanofiber group achieved a high apoptosis rate of 85.5%, comprising 53.3% late apoptotic (Q2) and 32.2% early apoptotic (Q3) cells. These results collectively demonstrate that the combination of US and AMF stimulation with 10 wt.% MPCs/PAN nanofibers significantly enhances tumor cell apoptosis, highlighting the superior therapeutic efficacy of this combinatorial approach for cancer treatment. This combinatorial approach led to pronounced inhibition of GBM cell migration and induction of apoptosis.

**Figure 6.**
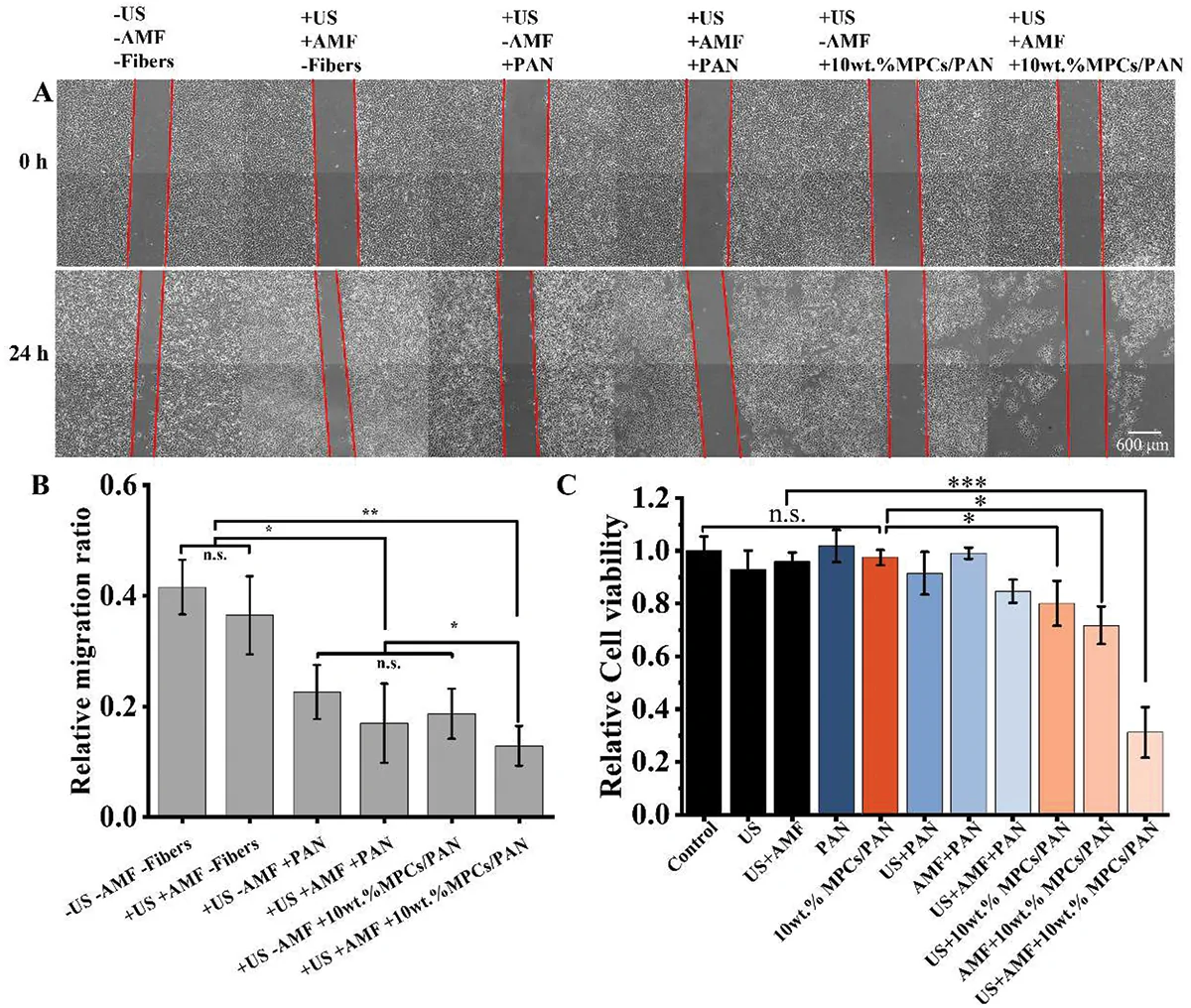
(A) Representative photographs of cell migration in six groups (control, US+AMF, US+PAN, US+AMF+PAN, US+10 wt.% MPCs/PAN, US+AMF+10 wt.% MPCs/PAN), (B) Quantitative evaluation of T98G cell migration rate following treatment under different stimulation conditions and co-cultured with nanofibers. The experimental groups include control, US+AMF, US+PAN, US+10 wt.% MPCs/PAN, US+AMF+PAN, and US+AMF+10 wt.% MPCs/PAN. Data obtained from three independent replicates (*n* = 3) were compared by one-way ANOVA with Tukey’s post hoc test, comparisons are annotated in the panels (*p < 0.05, **p < 0.01, ***p < 0.001; ns = not significant). (C) relative cell viability of control, US, US+AMF, PAN, 10 wt.% MPCs/PAN, US+PAN, AMF+PAN, US+AMF+PAN, US+10 wt.% MPCs/PAN, AMF +10 wt.% MPCs/PAN, and US+AMF+10 wt.% MPCs/PAN groups. Data obtained from three independent replicates (*n* = 3) were compared by one-way ANOVA with Tukey’s post hoc test, comparisons are annotated in the panels (*p < 0.05, **p < 0.01, ***p < 0.001; ns = not significant).

## 4. Conclusions

We developed a multifunctional MPCs/PAN nanofibers platform that combinatorially integrates ultrasound-activated TTFs therapy with magnetic hyperthermia for the treatment of glioblastoma. The incorporation of MPCs enhanced polarization and improved the piezoelectric response under ultrasound stimulation, while simultaneously enabling efficient localized heat generation. This dual functionality facilitated in situ electric field generation and thermal therapy within a single platform. In vitro evaluations demonstrated that the integrated system significantly suppressed GBM cell migration and achieved enhanced antitumor efficacy compared to individual treatment modalities. Collectively, this work presents a versatile and generalizable strategy for designing multifunctional nanocomposites that combine complementary physical stimuli. It provides a promising foundation for the development of non-pharmacological therapeutic approaches for aggressive and treatment-resistant cancers. Looking ahead, the flexible MPCs/PAN nanofibers could be implanted at the tumor resection cavity wall, enabling local coupling of ultrasound and AMF stimulation. Future investigations will focus on biodegradable piezoelectric polymers to improve long-term biocompatibility and advance clinical translatability.

## Supplementary Material

Supplemental Material

## Data Availability

The data supporting this article have been included as part of the supplementary information (SI). Supplementary information is available.
