# Supplementary material for: Dual-modal Fe-MOF-derived piezoelectric nanofibers enable combinatorial tumor suppression via tumor treating fields and magnetic hyperthermia
Source: Sci Technol Adv Mater. 2026 Sep 11;27(1):2705817. doi: 10.1080/14686996.2026.2705817 (PMC13629815; doi:10.1080/14686996.2026.2705817)
Supplement: Supplementary file 1 [file TSTA_A_2705817_SM3788.docx]

Supporting Information

**Dual-modal Fe-MOF-derived piezoelectric nanofibers enable combinatorial tumor suppression via tumor treating fields and magnetic hyperthermia**

Shiqin Dai^a,b^, Ahmed Nabil^a^, Mitsuhiro Ebara^* a,b,c,d^

^a^Research Center for Macromolecules and Biomaterials, National Institute for Materials Science (NIMS), 1-1 Namiki, Tsukuba, Ibaraki 305-0044, Japan

^b^Graduate School of Pure and Applied Sciences, University of Tsukuba, Tsukuba, Ibaraki 305-0006, Japan

^c^Department of Materials Science and Technology, Tokyo University of Science, Tokyo 125-8585, Japan

^d^Department of Biochemistry and Microbiology, Faculty of Pharmaceutical Sciences, Chulalongkorn University, Bangkok 10330, Thailand

Corresponding author:

Prof. Dr. Mitsuhiro Ebara

[**Ebara.Mitsuhiro@nims.go.jp**](mailto:Ebara.Mitsuhiro@nims.go.jp)

[**https://orcid.org/0000-0002-7906-0350**](https://orcid.org/0000-0002-7906-0350)

**Table of contents**

Figure S1. Dynamic light scattering (DLS) of (A)Fe-MOF and (B) MPCs.

Figure S2. Figure S2. Scanning electron microscopy (SEM) images and corresponding fiber diameter quantification of 5 wt.% MPCs/PAN, 10 wt.% MPCs/PAN, and 15 wt.% MPCs/PAN nanofibers at magnifications of 2 k × and 5 k ×.

Figure S3. The Magnetization-Magnetic Field (M-H) curve of MPCs nanoparticles calcined at 500 ℃ was measured using a physical property measurement system.

Figure S4. Optical microscopy of MPCs/PAN nanofibers cross-sections for thickness characterization.

Figure S5. Effect of medium thickness on local electric field intensity generated by 10 wt.% MPCs/PAN nanofibers.

Figure S6. Representative optical microscopy images of T98G glioblastoma cells following various treatments. Left column: bright-field, right column: fluorescence overlay. From top to bottom: -US -AMF, +US -AMF, -US +AMF, and +US +AMF of control group and 10 wt.% MPCs/PAN group.

Figure S7. Flow cytometric analysis of tumor cell apoptosis using Annexin V-FITC/PI double staining. Cells were treated with or without 10 wt.% MPCs/PAN nanofibers under three stimulation conditions: −US/−AMF, −US/+AMF, and +US/+AMF. Q1: necrotic cells, Q2: late apoptotic cells, Q3: early apoptotic cells, Q4: live cells.

**Figure S1. Dynamic light scattering (DLS) of (A)Fe-MOF and (B) MPCs.** DLS analysis showed that the Fe-MOF had a particle size of 123 nm before calcination at 500 ℃, which slightly decreased to 100 nm for the resulting MPCs, indicating that the framework largely maintained its structural integrity post-calcination.

**Figure S2. Scanning electron microscopy (SEM) images and corresponding fiber diameter quantification of 5 wt.% MPCs/PAN, 10 wt.% MPCs/PAN, and 15 wt.% MPCs/PAN nanofibers at magnifications of 2 k × and 5 k ×.** SEM micrographs at both 2k and 5k magnifications demonstrate that all samples exhibit uniform, bead-free fibrous morphologies. The incorporation of MPCs does not lead to substantial variation in the average diameter of PAN fibers, although slight fluctuations can be observed (n = 3).

**Figure S3. The Magnetization-Magnetic Field (M-H) curve of MPCs nanoparticles calcined at 500 ℃ was measured using a physical property measurement system.** The magnetic behavior of the prepared MPCs was examined at room temperature using vibrating sample magnetometry (VSM) at an applied field of 1 T. The nanoparticles displayed a saturation magnetization (Ms) of 21.97 emu g^-1^ at 1 T, and the corresponding H-M profile showed minimal hysteresis, indicating their superparamagnetic characteristics.

**Figure S4.** Optical microscopy of MPCs/PAN nanofibers cross-sections for thickness characterization.

**Figure S5**. **Effect of medium thickness on local electric field intensity generated by 10 wt.% MPCs/PAN nanofibers.** The field strength decreases from 2.7 V cm⁻¹ at 0 mm to approximately 1.4 V cm⁻¹ at 4 mm medium thickness. Despite this attenuation, the residual electric field remains within the established therapeutic window for tumor treating fields (TTFields) therapy (1–3 V cm^-1^), supporting the potential efficacy of the 10 wt.% MPCs/PAN nanofibers for effective tumor growth inhibition under physiologically relevant conditions. Error bars represent standard deviation (n = 3).

**Figure S6.** **Representative optical microscopy images of T98G glioblastoma cells following various treatments.** Left column: bright-field, right column: fluorescence overlay. From top to bottom: -US -AMF, +US -AMF, -US +AMF, and +US +AMF of control group and 10 wt.% MPCs/PAN group.

**Figure S7.** **Flow cytometric analysis of tumor cell apoptosis using Annexin V-FITC/PI double staining.** Cells were treated with or without 10 wt.% MPCs/PAN nanofibers under three stimulation conditions: −US/−AMF, −US/+AMF, and +US/+AMF. Q1: necrotic cells, Q2: late apoptotic cells, Q3: early apoptotic cells, Q4: live cells.
